# Commentary: Acupoint glucocorticoid injection combined with focused ultrasound for granulomatous lobular mastitis: a retrospective study

**DOI:** 10.3389/fsurg.2026.1890019

**Published:** 2026-08-11

**Authors:** Lixia Zhu, Shurong Shen

**Affiliations:** 1 Zhejiang Chinese Medical University, Hangzhou, China; 2 Wenzhou Central Hospital, Wenzhou, China

**Keywords:** acupoint injection, focused ultrasound, glucocorticoid, granulomatous lobular mastitis, minimally invasive therapy, traditional Chinese medicine

## 1 Introduction

We read with great interest the retrospective study by Wang et al. (1) evaluating acupoint glucocorticoid injection combined with focused ultrasound for granulomatous lobular mastitis (GLM). The authors should be commended for addressing a clinically challenging condition. GLM is a benign inflammatory breast disease that predominantly affects women of reproductive age and may cause pain, abscesses, sinus tracts, recurrence, cosmetic disfigurement, and psychological distress (2, 3). Existing treatment strategies—including observation, systemic corticosteroids, intralesional corticosteroid injection, immunosuppressive therapy, antibiotics, and surgery—remain heterogeneous, and recurrence continues to pose a substantial clinical challenge (4, 5). The study by Wang et al. is clinically appealing in that it proposes an integrative, breast-conserving strategy combining local glucocorticoid delivery with high-intensity focused ultrasound (HIFU). The reported outcomes—higher complete response rates, shorter remission times, lower recurrence, improved cosmetic results, and fewer systemic adverse events—suggest that this combined approach may serve as a promising adjunctive option. Nevertheless, several methodological, technical, and mechanistic considerations warrant further discussion.

## 2 Methodological and technical considerations

A central concern lies in therapeutic attribution. The combined intervention encompasses several potentially active components: acupoint injection, local corticosteroid delivery, repeated procedural contact, individualized traditional Chinese medicine (TCM) syndrome differentiation, and HIFU. Because Wang et al. (1) compared the combined intervention against conventional intralesional corticosteroid injection and oral corticosteroids, the design cannot determine whether the observed benefit derived from acupoint specificity, local steroid pharmacology, HIFU, or a synergistic interaction. This distinction carries clinical relevance, since intralesional corticosteroid therapy alone has been reported as effective for GLM with reduced systemic exposure relative to oral corticosteroids (6). Image-guided thermal or mechanical interventions, such as HIFU and microwave ablation, may theoretically modify tissue architecture, inflammatory lesion resorption, tissue permeability, local vascularity, and drug penetration, although direct evidence in GLM remains limited (7, 8). Future investigations would benefit from incorporating component-control arms—such as acupoint corticosteroid injection alone, HIFU alone, non-acupoint local corticosteroid injection plus HIFU, and standard intralesional corticosteroid injection—to clarify whether acupoints confer benefits beyond regional drug delivery.

The authors selected acupoints, including Rugen (ST18) and Qimen (LR14), according to TCM theory. Although this individualized approach holds clinical meaning within integrative medicine, it complicates reproducibility and mechanistic interpretation. As these points lie anatomically close to the breast and chest wall, part of the therapeutic effect may theoretically be explained by regional corticosteroid delivery, local neuroimmune modulation, or microcirculatory changes rather than meridian specificity *per se*, although such mechanisms require formal verification. Trials employing sham acupoints, non-acupoint injection sites, or standardized vs. individualized protocols may help disentangle pharmacological delivery, needle stimulation, and syndrome-based selection. Adherence to the revised Standards for Reporting Interventions in Clinical Trials of Acupuncture (STRICTA) and the SPIRIT-TCM Extension 2018 would further enhance transparency and protocol fidelity (9).

A technical inconsistency also warrants clarification: the Methods section of Wang et al.(1) specifies a frequency of 0.98 MHz, whereas the Discussion mentions 20 MHz. Although this likely reflects a typographical error, the distinction is technically consequential, since ultrasound frequency substantially influences penetration depth, focal energy deposition, and safety. A 20 MHz frequency would generally imply considerably shallower penetration than a sub-megahertz therapeutic system. We respectfully suggest that the authors clarify this point in a corrigendum and provide a comprehensive parameter table including frequency, acoustic power, spatial-peak temporal-average intensity (I_SPTA), duty cycle, focal depth, focal volume, sonication duration, total acoustic dose, and skin-cooling procedures. Furthermore, the original report attributes the mechanism to both coagulative necrosis and sub-ablative effects (cavitation, microstreaming, increased permeability); extrapolating from general HIFU mechanisms (8), these represent distinct therapeutic modes, and clarifying the intended biological effect would strengthen technical interpretation.

## 3 Bias, outcomes, and future directions

The retrospective, non-randomized, open-label design constitutes an important limitation, as treatment allocation depended on clinician judgment and patient preference. Even when baseline characteristics appear balanced, unmeasured confounders—including lesion complexity, abscess or sinus burden, microbiological status, prior antibiotic exposure, endocrine factors, and adherence—may have influenced outcomes. The reported efficacy data, while clinically impressive, warrant cautious interpretation. Wang et al. (1) reported complete response rates of 92.2% in the combined group, 75.4% in the conventional intralesional corticosteroid injection group, and 68.9% in the oral corticosteroid group among 186 patients with the follow-up duration described in the original report. Patients who switched modalities or required rescue therapy were excluded to preserve group integrity; however, treatment switching and rescue therapy serve as critical indicators of inadequate response. Future studies should adopt intention-to-treat analysis and incorporate rescue therapy, treatment switching, recurrence, and failure to achieve complete response as composite outcomes. Cosmetic and quality-of-life outcomes are particularly salient, as GLM frequently affects young women and surgery may result in scarring, asymmetry, and breast deformity (3, 5). However, subjective endpoints are susceptible to expectation and observer bias in open-label studies. Standardized photography, blinded independent cosmetic assessment, validated patient-reported outcome measures such as the BREAST-Q, and extended follow-up would substantially reinforce the interpretation of cosmetic benefit (10).

## 4 Discussion

The work of Wang et al. (1) presents an innovative and clinically relevant strategy for GLM that may reduce systemic corticosteroid exposure while preserving breast appearance. Its most valuable contribution may lie in emphasizing local inflammatory microenvironment remodeling—suggesting that GLM management may be approached not only through systemic immunosuppression, but also through localized anti-inflammatory delivery, image-guided tissue modulation, and potentially neuroimmune regulation. Nonetheless, given the retrospective design, absence of component-control arms, limited mechanistic data, and incomplete technical reporting, the findings should be regarded as hypothesis-generating rather than definitive. Prospective, multicenter, randomized trials are warranted. Future studies should incorporate standardized HIFU reporting, rigorous component-control arms, pre-registered TCM intervention rules, intention-to-treat analysis, blinded outcome assessment, validated patient-reported outcomes, and extended follow-up. Clinically relevant endpoints may include complete response, time to remission, recurrence, need for rescue therapy or treatment switching, adverse events, cosmetic outcomes, quality of life, and imaging-based lesion volume change. In conclusion, acupoint glucocorticoid injection combined with HIFU represents a promising breast-conserving approach for GLM, and further rigorous investigation is required to elucidate its specific mechanisms, reproducibility, comparative effectiveness, and long-term safety.

## References

[1] Wang H Ning W Wang H Li H Zhang G. Acupoint glucocorticoid injection combined with focused ultrasound for granulomatous lobular mastitis: a retrospective study. Front Surg. (2026) 13:1715357. 10.3389/fsurg.2026.1715357 41960207 PMC13056661

[2] Yuan Q-Q Xiao S-Y Farouk O Du Y-T Sheybani F Tan QT. Management of granulomatous lobular mastitis: an international multidisciplinary consensus (2021 edition). Mil Med Res. (2022) 9(1):20. 10.1186/s40779-022-00380-5 35473758 PMC9040252

[3] Zhou Y Xu L. Clinical efficacy of different methods for treatment of granulomatous lobular mastitis: a systematic review and network meta-analysis. PLoS One. (2025) 20(2):e0318236. 39899590 10.1371/journal.pone.0318236 PMC11790104

[4] Martinez-Ramos D Simon-Monterde L Suelves-Piqueres C Queralt-Martin R Granel-Villach L Laguna-Sastre JM. Idiopathic granulomatous mastitis: a systematic review of 3060 patients. Breast J. (2019) 25(6):1245–50. 10.1111/tbj.13446 31273861

[5] Lei X Chen K Zhu L Song E Su F Li S. Treatments for idiopathic granulomatous mastitis: systematic review and meta-analysis. Breastfeed Med: Off J Acad Breastfeed Med. (2017) 12(7):415–21. 10.1089/bfm.2017.0030 28731822

[6] Toktas O Konca C Trabulus DC Soyder A Koksal H Karanlik H. A novel first-line treatment alternative for noncomplicated idiopathic granulomatous mastitis: combined i˙ntralesional steroid i˙njection with topical steroid administration. Breast Care (Basel). (2021) 16(2):181–7. 10.1159/000507951 34012373 PMC8114037

[7] Lin L Zheng Z Zhang J Liu X Chen DR Wang H. Treatment of idiopathic granulomatous mastitis using ultrasound-guided microwave ablation: a report of 50 cases. Int J Hyperth: Off J Eur Soc Hyperthermic Oncol N Am Hyperth Group. (2021) 38(1):1242–50. 10.1080/02656736.2021.1965225 34402370

[8] Bachu VS Kedda J Suk I Green JJ Tyler B. High-intensity focused ultrasound: a review of mechanisms and clinical applications. Ann Biomed Eng. (2021) 49(9):1975–91. 10.1007/s10439-021-02833-9 34374945 PMC8608284

[9] Dai L Cheng C- Tian R Zhong LLD Li Y- Lyu A- Standard protocol items for clinical trials with traditional Chinese medicine 2018: recommendations, explanation and elaboration (SPIRIT-TCM extension 2018). Chin J Integr Med. (2019) 25(1):71–9. 10.1007/s11655-018-2999-x 30484022

[10] Pusic AL Klassen AF Scott AM Klok JA Cordeiro PG Cano SJ. Development of a new patient-reported outcome measure for breast surgery: the BREAST-Q. Plast Reconstr Surg. (2009) 124(2):345–53. 10.1097/PRS.0b013e3181aee807 19644246

